# Epidemiology and outcomes of hyponatremia in patients with COVID-19—A territory-wide study in Hong Kong

**DOI:** 10.3389/fmed.2022.1096165

**Published:** 2023-01-11

**Authors:** Gordon Chun Kau Chan, Chun Ka Wong, Benjamin Y. F. So, Jack Kit Chung Ng, Grace Chung Yan Lui, Cheuk Chun Szeto, Ivan Fan Ngai Hung, Hung Fat Tse, Sydney C. W. Tang, Tak Mao Chan, Kai Ming Chow, Desmond Y. H. Yap

**Affiliations:** ^1^Division of Nephrology, Department of Medicine and Therapeutics, Prince of Wales Hospital, The Chinese University of Hong Kong, Shatin, Hong Kong SAR, China; ^2^Division of Cardiology, Department of Medicine, Queen Mary Hospital, The University of Hong Kong, Pokfulam, Hong Kong SAR, China; ^3^Division of Nephrology, Department of Medicine, Queen Mary Hospital, The University of Hong Kong, Pokfulam, Hong Kong SAR, China; ^4^Division of Infectious Diseases, Department of Medicine and Therapeutics, Prince of Wales Hospital, The Chinese University of Hong Kong, Shatin, Hong Kong SAR, China; ^5^Division of Infectious Diseases, Department of Medicine, Queen Mary Hospital, The University of Hong Kong, Pokfulam, Hong Kong SAR, China

**Keywords:** COVID-19, epidemiology, hyponatremia, outcomes, sodium

## Abstract

**Background:**

Hyponatremia is common in COVID-19, but its epidemiology and impact on clinical outcomes in relation to different variants, especially the Omicron variant, requires further clarification.

**Methods:**

This was a territory-wide retrospective study to investigate the epidemiology and outcomes of COVID-19 patients with hyponatremia from January 1, 2020 to March 31, 2022 in Hong Kong. The primary outcome was 30-day mortality of patients with COVID-19 and hyponatremia at presentation. Secondary outcomes included rate of hospitalization, intensive care unit (ICU) hospitalization, overall duration of hospitalization, and duration of ICU hospitalization.

**Results:**

A total of 53,415 COVID-19 patients were included for analysis, of which 14,545 (27.2%) had hyponatremia at presentation. 9813 (67.5%), 2821 (19.4%), and 1911 (13.1%) had mild (130 to <135 mmol/L), moderate (125 to <130 mmol/L), and severe hyponatremia (<125 mmol/L) at presentation respectively. Age, male sex, diabetes, active malignancy, white cell count, serum creatinine, hypoalbuminemia, C-reactive protein, and viral loads were independent predictors for hyponatremia in COVID-19 patients (*P* < 0.001, for all). Hyponatremic patients had increased 30-day mortality (9.7 vs. 5.7%, *P* < 0.001), prolonged hospitalization (11.9 ± 15.1 days vs. 11.5 ± 12.1 days, *P* < 0.001), and more ICU admissions (7.0% vs. 3.3%, *P* < 0.001). Patients diagnosed during the “fifth wave” Omicron BA.2 outbreak had 2.29-fold risk (95% CI 2.02–2.59, *P* < 0.001) of presenting with hyponatremia compared to other waves.

**Conclusion:**

Hyponatremia is common among COVID-19 patients, and may serve as a prognostic indicator for unfavorable outcomes and increased healthcare utilization in the evolving COVID-19 outbreak.

## Introduction

Hyponatremia is the most common electrolyte disorder among inpatients, and is classically associated with community-acquired pneumonia (CAP) (1–3). Moderate-to-severe hyponatremia *per se* may cause permanent neurological damage, and hyponatremia is also correlated with higher levels of inflammatory markers, mortality rates, and other indices of disease severity in a variety of conditions (4–10). Furthermore, the treatment of moderate-to-severe hyponatremia often requires close inpatient monitoring, as neurological sequelae may arise from inappropriate rates of correction of blood sodium levels, causing significant stress on scarce healthcare resources (11, 12).

An association between COVID-19 and hyponatremia is well-recognized, with studies reporting incidences of hyponatremia of up to 25% (13–20). Hyponatremia was associated with severity of pulmonary infiltrates, need for intubation and mechanical ventilation, and death. In addition, at least two different studies have correlated hyponatremia in COVID-19 with elevated IL-6 levels, which could theoretically stimulate anti-diuretic hormone (ADH) release from the pituitary (19, 21–23). Treatment with the IL-6-specific monoclonal antibody tocilizumab normalized sodium levels in patients with COVID-19, in conjunction with overall disease improvement (21).

Interestingly, hyponatremia often manifests as part of a specific clinical syndrome in CAP. Some bacterial infections are more associated with hyponatraemia, such as pneumonia caused by *Legionella pneumophila* (legionellosis), suggesting that some infections stimulate ADH release or other neurohormonal pathways more potently than others (24, 25). Similarly, the common clinical manifestations of COVID-19 have evolved during the pandemic depending on the dominant circulating variant at the time (26). Since all the reported studies so far were performed in the pre-Omicron era, we postulated that the epidemiology and clinical correlates of COVID-19-related hyponatremia might be different during the global Omicron outbreak. Here, we report on the territory-wide prevalence and clinical outcomes of COVID-19 patients with hyponatremia in Hong Kong, focusing on a major outbreak of the SARS-CoV-2 Omicron BA.2 subvariant in early 2022 (the “fifth wave” in Hong Kong). We also compared these findings with earlier outbreaks of COVID-19 due to other variants, as well as with the outbreak of Severe Acute Respiratory Syndrome (SARS) due to SARS-CoV-1 in 2003.

## Materials and methods

### Study design and patients’ selection

This was a territory-wide retrospective observational cohort study. The study protocol was approved by the Institutional Review Board of the University of Hong Kong/Hospital Authority Hong Kong West Cluster (HKU/HA IRB UW 13-625) and the requirement for informed consent was waived. The study was conducted in compliance with the Declaration of Helsinki.

Adult patients who first tested positive for SARS-CoV-2 reverse transcription-polymerase chain reaction (RT-PCR) in respiratory samples, and with serum sodium level available on the same day from January 1, 2020 to March 31, 2022, were identified from the Clinical Data Analysis and Reporting System (CDARS) database of the Hong Kong Hospital Authority. All patients with COVID-19 requiring hospitalization were admitted to public hospitals. Treatment of patients with COVID-19 was at clinicians’ discretion and according to prevailing protocols at the time. Retrieved data included patients’ demographics, diagnoses, prescriptions, laboratory results, and death. Data retrieved were de-identified to ensure patient confidentiality. Disease diagnoses were coded and analyzed according to ICD-9-CM (Supplementary Table 1). The estimated glomerular filtration rate (eGFR) was calculated by the CKD Epidemiology Collaboration (CKD-EPI) creatinine equation (27). Hyponatremia was defined by serum sodium below 135 mmol/L, which were mostly measured by the indirect ion selective electrode (ISE) methods (28). Patients were further classified into mild, moderate, and severe hyponatremia if their serum sodium level ranged from 130 to <135 mmol/L, 125 to <130 mmol/L, and <125 mmol/L, respectively. The volume status of patients with hyponatremia was further evaluated by the percentage change of serum creatinine from hyponatremia to eunatremia as described by Ruiz-Sánchez et al. (29). In essence, a percentage change of creatinine above 10% indicates hypovolemic status, while a change below −3% indicates euvolemic status. For comparison with hospitalized COVID-19 patients, we also retrieved clinical data of patients diagnosed with SARS [defined as positivity for Severe Respiratory Syndrome (SRS) agent, later renamed SARS-CoV-1, by RT-PCR from respiratory samples] during the period from January 1, 2002 to December 31, 2003.

### Outcomes

All subjects were followed for at least 90 days or until death. The primary outcome was 30-day mortality following diagnosis of COVID-19. The secondary outcomes included 90-day mortality, rate of hospitalization, intensive care unit (ICU) hospitalization, overall duration of hospitalization, and duration of ICU hospitalization. We also compared the rate of hyponatremia among local waves dominated by different SARS-CoV-2 variants (Supplementary Table 2).

### Statistical analysis

Statistical analysis was performed by SPSS for Mac software version 27.0 (IBM corporation, Armonk, NY, USA). Continuous data was expressed as mean ± standard deviation, while categorical data was presented as number (percentage). Patients were grouped according to the presence/absence of hyponatremia at presentation for analysis. Data was compared between groups by chi-square test, student’s *t*-test, or Mann–Whitney *U* test as appropriate. Time-to-event analysis was performed for the primary outcome by the Kaplan–Meier method and compared by the log-rank test. Multivariate logistic and Cox proportional hazard regression analyses were further performed to adjust for confounders. Factors which are known risk factors, and parameters with *p*-value below 0.1 in the univariate model were added into the multivariate analyses. These factors include age, gender, SARS-CoV-2 PCR CT-value, hemoglobin, white blood cell, eGFR, albumin, C-reactive protein, D-dimer levels in blood, co-existing diabetes mellitus, hypertension, ischemic heart disease, congestive heart failure, cardiac arrhythmia, cerebrovascular accident, chronic obstructive airway disease, malignancy, dementia, and chronic liver disease. *P*-values below 0.05 were considered statistically significant. All probabilities were two-tailed.

## Results

### Patient characteristics

**FIGURE 1:**
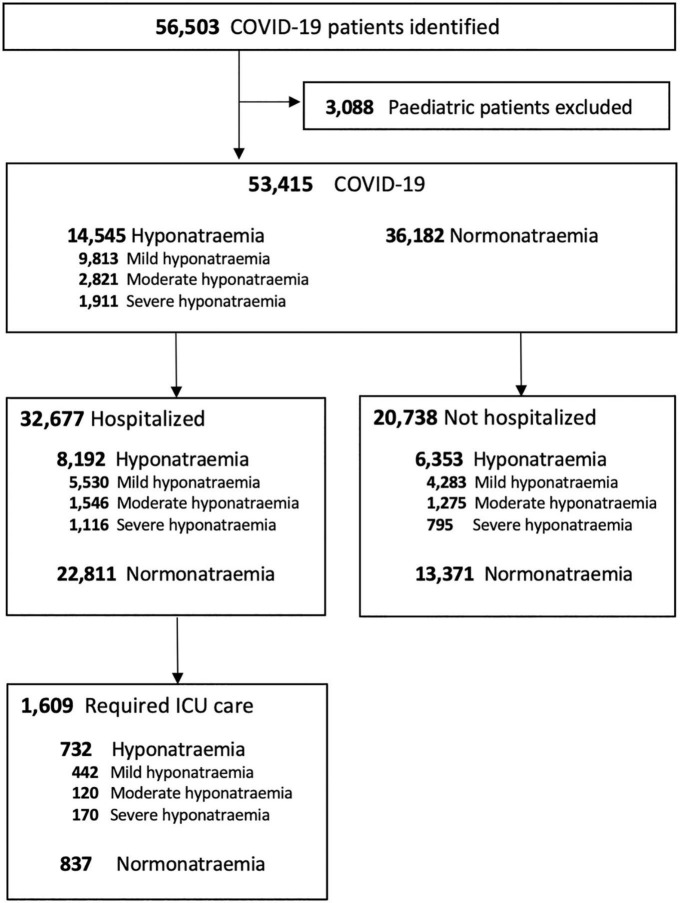
Flow chart of COVID-19 patients.

Data from a total of 53,415 adult COVID-19 patients were retrieved and included for final analysis (Figure 1). 14,545 (27.2%) COVID-19 patients developed hyponatremia on presentation. Among hyponatremic COVID-19 patients, 9,813 (67.5%), 2,821 (19.4%), and 1,911 (13.1%) had mild, moderate, and severe hyponatremia, respectively.

The clinical characteristics of COVID-19 patients with or without hyponatremia at presentation are summarized in Table 1. COVID-19 patients with hyponatremia on presentation were older (74.3 ± 16.1 years vs. 62.4 ± 22.0 years, *P* < 0.001) and showed male predominance (56.6 vs. 50%, *P* < 0.001). Patients with hyponatremia at presentation had higher prevalence of various comorbidities including but not limited to diabetes mellitus (DM), hypertension (HT), ischemic heart disease (IHD), chronic kidney disease (CKD), dementia, and malignancy (*P* < 0.01 for all). Hyponatremic patients presented with higher white cell counts, but lower lymphocyte counts and hemoglobin levels (*P* < 0.001, for all). eGFR (65.2 ± 31.1 vs. 79.7 ± 31.1 ml/min, *P* < 0.001) and serum albumin (32.9 ± 6.4 vs. 36.8 ± 6.4 g/L, *P* < 0.001) were lower in COVID-19 patients with hyponatremia. COVID-19 patients with hyponatremia also had higher SARS-CoV-2 viral loads (Ct values 22.2 ± 6.4 vs. 23.5 ± 6.8, *P* < 0.001), C-reactive protein (CRP) (6.7 ± 7.1 vs. 3.7 ± 5.9 mg/L, *P* < 0.001), creatine kinase (410 ± 1491 vs. 250 ± 1599 U/L, *P* < 0.001), and D-dimer levels (1284 ± 1871 vs. 862 ± 1567 ng/mL, *P* < 0.001) than those without hyponatremia (Tables 1, 2). By evaluating the percentage change of serum creatinine from hyponatremia to eunatremia, 53.9% of patients were hypovolemic.

**TABLE 1:** Clinical characteristics of COVID-19 patients with or without hyponatremia at presentation.

	Hyponatremia (*n* = 14,545)	Normonatremia (*n* = 36,182)	*P*-value
Age	74.3 ± 16.1	62.4 ± 22.0	<0.001^a^
Age older than 65, No. (%)	10,887 (74.9%)	17,679 (48.9%)	<0.001^b^
Male, No. (%)	8,237 (56.6%)	18,103 (50.0%)	<0.001^b^
**Comorbidities**			
Diabetes mellitus	4,968 (34.2%)	7,184 (19.9%)	<0.001^b^
Hypertension	7,910 (54.4%)	13,618 (37.6%)	<0.001^b^
Ischemic heart disease	2,245 (15.4%)	3,724 (10.3%)	<0.001^b^
Cerebrovascular accident	1,826 (12.6%)	2,847 (7.9%)	<0.001^b^
Cardiac arrhythmia	2,241 (15.4%)	3,858 (10.7%)	<0.001^b^
Congestive heart failure	1,472 (10.1%)	2,676 (7.4%)	<0.001^b^
Chronic obstructive airway disease	954 (6.6%)	1,604 (4.4%)	<0.001^b^
Asthma	279 (1.9%)	593 (1.6%)	=0.03^b^
Pneumoconiosis	201 (1.4%)	242 (0.7%)	<0.001^b^
Dementia	1,732 (11.9%)	3,380 (9.3%)	<0.001^b^
Chronic liver disease	1,033 (7.1%)	2,026 (5.6%)	<0.001^b^
Active malignancy	3,003 (20.6%)	5,517 (15.2%)	<0.001^b^
Chronic kidney disease (eGFR < 90 ml/min)	12,004 (82.5%)	24,145 (66.7%)	<0.001^b^
eGFR 60–90 ml/min (Stage 2)	7,304 (50.2%)	16,915 (46.7%)	
eGFR 30–59 ml/min (Stage 3)	2,869 (19.7%)	4,860 (13.4%)	
eGFR 15–29 ml/min (Stage 4)	915 (6.3%)	1,435 (4.0%)	
eGFR <15 ml/min (Stage 5)	916 (6.3%)	934 (2.6%)	
**Laboratory parameters**			
Hemoglobin (g/dL)	11.9 ± 2.2	12.8 ± 2.2	<0.001^a^
White cell count (10^9^/L)	8.7 ± 7.0	7.0 ± 4.0	<0.001^a^
Neutrophil (10^9^/L)	6.5 ± 4.7	4.9 ± 3.4	<0.001^a^
Lymphocyte (10^9^/L)	1.0 ± 1.9	1.3 ± 1.0	<0.001^a^
Neutrophil to lymphocyte ratio	9.1 ± 10.2	5.6 ± 7.2	<0.001^a^
Platelet (10^9^/L)	221 ± 102	223 ± 88	0.05^a^
Sodium (mmol/L)	129.9 ± 5.6	138.6 ± 2.3	<0.001^a^
Potassium (mmol/L)	4.0 ± 0.7	3.9 ± 0.5	<0.001^a^
Urea (mmol/L)	8.8 ± 8.0	6.7 ± 6.1	<0.001^a^
Creatinine (umol/L)	144 ± 205	103 ± 124	<0.001^a^
eGFR (by CKD-EPI equation)	65.2 ± 31.1	79.7 ± 31.1	<0.001^a^
Albumin (g/L)	32.9 ± 6.4	36.8 ± 6.4	<0.001^a^
C-reactive protein (mg/L)	6.7 ± 7.1	3.7 ± 5.9	<0.001^a^
Calcium (mmol/L)	2.16 ± 0.17	2.23 ± 0.15	<0.001^a^
Phosphate (mmol/L)	1.11 ± 0.49	1.09 ± 0.37	<0.001^a^
Osmolality (mOsm/kg)	269 ± 22	302 ± 33	<0.001^a^
Thyroid stimulating hormone (mIU/L)	1.6 ± 3.6	1.7 ± 3.8	0.5^a^
Creatine kinase (U/L)	410 ± 1491	250 ± 1599	<0.001^a^
D-dimer (ng/ml)	1284 ± 1871	862 ± 1567	<0.001^a^
Urine sodium (mmol/L)	49.0 ± 34.5	50.3 ± 40.7	0.6^a^
Urine osmolality	407 ± 165	438 ± 196	0.01^a^

Data are presented as mean ± standard deviation unless specified and compared by Student’s *t*-test^a^ and chi-square test^b^. COVID-19, coronavirus disease-2019; SARS-CoV-2, severe acute respiratory syndrome coronavirus 2; RT-PCR, reverse transcription polymerase chain reaction; Ct value, cycle threshold value; eGFR, estimated glomerular filtration rate; CKD-EPI, chronic kidney disease epidemiology collaboration.

**TABLE 2:** The rates of hospitalization and intensive care unit admission in COVID-19 patients with or without hyponatremia at presentation and the relationships with different local waves of outbreak.

	Hyponatremia (*n* = 14,545)	Normonatremia (*n* = 36,182)	*P*-value
SARS-CoV-2 RT-PCR Ct value	22.2 ± 6.4	23.5 ± 6.8	<0.001^a^
Hospitalized	8,472 (58.2%)	23,169 (64.0%)	<0.001^b^
Hospitalization period	11.9 ± 15.1	11.5 ± 12.1	0.005^a^
Prolonged hospitalization (%, among hospitalized)	2,317 (27.3%)	5,951 (25.7%)	0.003^b^
Non-hospitalized	6,073 (41.8%)	13,013 (36.0%)	<0.001^b^
Required ICU admission	1,012 (7.0%)	1,195 (3.3%)	0.001^b^
ICU hospitalization period	7.1 ± 12.4	8.2 ± 13.1	0.04^a^
Prolonged ICU hospitalization (%, among hospitalized in ICU)	256 (25.3%)	356 (29.8%)	0.02^b^
Died in 30 days	1,407 (9.7%)	2,051 (5.7%)	0.001^b^
Local wave (Time periods; predominant SARS-CoV-2 variants)			<0.001^b^
Second wave [1st to 30th April 2020; D614G (38)]	59 (7.3%)	746 (92.7%)	
Third wave [15th Jun–30th September 2020; B.1.1.63 (39)]	309 (9.9%)	2,808 (90.1%)	
Fourth wave [November 1, 2020–February 28, 2021; B.1.36.27 (39)]	562 (11.1%)	4,514 (88.9%)	
Fifth wave (1st Jan–31st Mar 2022; Omicron BA.2)	13,530 (33.8%)	26,484 (66.2%)	

Data are presented as mean ± standard deviation unless specified and compared by Student’s *t*-test^a^ and chi-square test^b^. COVID-19, coronavirus disease-2019; ICU, intensive care unit; VOC, variants of concern.

### Predictors of hyponatremia in COVID-19 patients

Multivariate analysis showed that older age, male gender, comorbidities such as DM, active malignancy, white cell count, serum creatinine, albumin, and CRP were independent predictors of hyponatremia in patients with COVID-19 (*P* < 0.001, for all) (Supplementary Table 7).

### Hospitalization and ICU admission

32,677 (61.2%) COVID-19 patients were hospitalized. Patients with hyponatremia at presentation had longer overall hospital stays (11.9 ± 15.1 vs. 11.5 ± 12.1 days, *P* < 0.001). Hyponatremic patients also had higher rates of ICU admission (7.0 vs. 3.3%, *P* < 0.001), though ICU stays were shorter (7.1 ± 12.4 vs. 8.2 ± 13.1 days, *P* = 0.004). Hyponatremia was more common in COVID-19 patients requiring ICU admission than those who did not (45.5 vs. 24.0%, *P* < 0.001). Blood sodium levels at presentation were significantly lower in those who required ICU admission (133.6 ± 9.0 vs. 137.3 ± 6.4 mmol/L, *P* < 0.001) (Supplementary Table 3). Multivariate analysis showed that hyponatremia of any extent was associated with increased risk for ICU admission in COVID-19 patients (*P* < 0.05, for any degree of hyponatremia), and the effect was especially marked in patients with severe hyponatremia (OR 4.42, 95% CI 3.35–5.82, *P* < 0.001) (Table 3). Age, male sex, viral load, DM, dementia, low hemoglobin, white cell count, serum creatinine, CRP, and D-dimer levels at presentation were independent risk factors for ICU admission in COVID-19 patients (*P* < 0.05, for all).

**TABLE 3:** Predictors of intensive care unit admission in patients with COVID-19.

	Univariate model		Multivariate model	
	**OR (95% CI)**	***P*-value**	**OR (95% CI)**	***P*-value**
**Severity of hyponatremia**				
Mild	2.28 (2.02–2.57)	<0.001	1.83 (1.52–2.21)	<0.001
Moderate	2.21 (1.81–2.70)	<0.001	1.38 (1.01–1.89)	0.04
Severe	4.72 (3.95–5.63)	<0.001	4.42 (3.35–5.82)	<0.001
**Demographics**				
Age	1.004 (1.002–1.006)	0.001	0.99 (0.98–0.99)	<0.001
Male sex	1.75 (1.58–1.94)	<0.001	1.42 (1.20–1.69)	<0.001
**Comorbidities**				
Diabetes mellitus	1.94 (1.75–2.16)	<0.001	1.52 (1.27–1.83)	<0.001
Hypertension	1.52 (1.38–1.68)	<0.001		
Ischemic heart disease	1.45 (1.26–1.67)	<0.001		
Active malignancy	1.67 (1.48–1.88)	<0.001		
Dementia	0.26 (0.20–0.35)	<0.001	0.18 (0.11–0.29)	<0.002
Congestive heart failure	1.40 (1.19–1.64)	<0.001		
Arrhythmia	1.37 (1.19–1.58)	<0.001		
Chronic liver disease	1.70 (1.42–2.02)	<0.001		
**Laboratory parameters**				
SARS-CoV-2 PCR Ct value	0.97 (0.96–0.98)	<0.001	0.97 (0.95–0.98)	<0.001
Hemoglobin	0.92 (0.90–0.94)	<0.001	1.06 (1.01–1.10)	0.01
White cell count	1.04 (1.04–1.05)	<0.001	1.02 (1.00–1.03)	0.009
Creatinine (for every 100 μmol/L rise)	1.14 (1.11–1.17)	<0.001	1.08 (1.04–1.75)	<0.001
Albumin	0.94 (0.93–0.94)	<0.001		
C-reactive protein	1.06 (1.05–1.07)	<0.001	1.04 (1.03–1.05)	<0.001
D-dimer (for every 1000 ng/ml rise)	1.14 (1.11–1.17)	<0.001	1.05 (1.01–1.09)	0.02

COVID-19, coronavirus disease-2019; CI, confidence interval; SARS-CoV-2, severe acute respiratory syndrome coronavirus 2; OR, odds ratio; RT-PCR, reverse transcription polymerase chain reaction.

### Mortality

**FIGURE 2:**
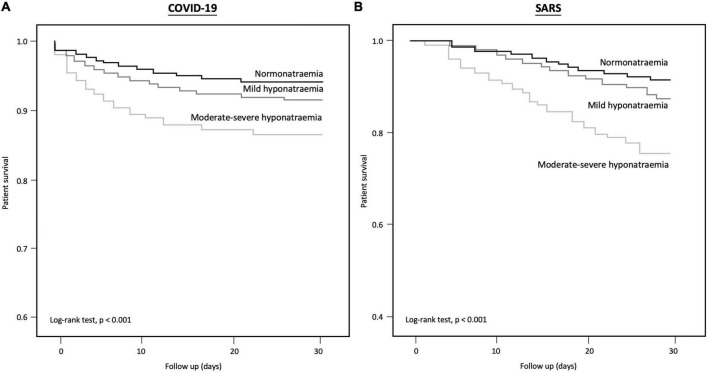
Kaplan–Meier curve for 30-day survival in **(A)** COVID-19, and **(B)** SARS patients with normo-, mild hypo-, and moderate-severe hyponatraemia.

COVID-19 patients with moderate-to-severe hyponatremia had higher mortality than those with normonatremia or mild hyponatremia (log-rank test: *P* < 0.001) (Figure 2; Supplementary Figure 1). In total, 4,318 (8.1%) COVID-19 patients died within 30 days (mean survival 7.4 ± 7.9 days), including 9.7% of all hyponatremic patients and 5.7% of normonatremic patients (Supplementary Table 5). The 30-day survival rates for normonatremic, mild, moderate, and severe hyponatremic patients were 94.3, 92.0, 87.2, and 86.5%, respectively (Figure 2), whereas the 90-day survival rates for normonatremic, mild, moderate, and severe hyponatremic patients were 94.2, 91.9, 87.0, and 86.5%, respectively (Supplementary Figure 1). On multivariate analysis, both moderate and severe hyponatremia were predictive of 30-day mortality in COVID-19 patients [adjusted hazard ratio (aHR) 1.24 and 1.71; 95% CI 1.04–1.48 and 1.4–2.09; *P* = 0.02 and < 0.001, respectively] (Table 4). Other independent predictors for 30-day mortality in COVID-19 patients included age, male sex, viral load, cardiovascular accident (CVA), chronic obstructive airway disease (COAD), arrhythmia, low hemoglobin, low eGFR, hypoalbuminemia, white cell count, CRP, and D-dimer levels (*P* < 0.05, for all). Furthermore, the multivariate Cox regression showed that euvolemic hyponatremia but not hypovolemic hyponatraemia (both evaluated by the percentage change of serum creatinine from hyponatremia to eunatremia) was predictive of 90-day mortality (AHR 2.03 and 1.16, respectively, 95% CI 1.77–2.32 and 0.99–1.37, *P* < 0.001 and 0.06).

**TABLE 4:** Risk factors for 30-day mortality in patients with COVID-19.

	Univariate model		Multivariate model	
	**HR (95% CI)**	***P*-value**	**Adjusted HR (95% CI)**	***P*-value**
**Severity of hyponatremia**				
Mild	1.56 (1.44–1.70)	<0.001		
Moderate	2.76 (2.47–3.09)	<0.001	1.24 (1.04–1.48)	0.02
Severe	3.02 (2.65–3.44)	<0.001	1.71 (1.40–2.09)	<0.001
**Demographics**				
Age	1.06 (1.06–1.06)	<0.001	1.03 (1.03–1.04)	<0.001
Male sex	1.35 (1.27–1.43)	<0.001		
**Comorbidities**				
Diabetes mellitus	1.71 (1.61–1.83)	<0.001		
Hypertension	2.37 (2.22–2.52)	<0.001		
Ischemic heart disease	1.97 (1.83–2.12)	<0.001		
Cerebrovascular accident	2.31 (2.15–2.50)	<0.001	1.19 (1.04–1.36)	0.01
COAD	2.07 (1.87–2.29)	<0.001	1.51 (1.27–1.81)	<0.001
Active malignancy	1.56 (1.45–1.68)	<0.001		
Dementia	2.62 (2.45–2.81)	<0.001		
Congestive heart failure	2.48 (2.29–2.68)	<0.001		
Arrhythmia	2.28 (2.13–2.45)	<0.001	1.23 (1.08–1.41)	0.002
Chronic liver disease	1.50 (1.35–1.67)	<0.001		
**Laboratory parameters**				
SARS-CoV-2 PCR Ct value	0.97 (0.96–0.97)	<0.001	0.98 (0.97–0.99)	<0.001
Hemoglobin	0.79 (0.78–0.80)	<0.001	0.97 (0.94–0.99)	0.01
White cell count	1.02 (1.02–1.02)	<0.001	1.02 (1.01–1.03)	<0.001
eGFR (by CKD-EPI equation)	0.97 (0.97–0.97)	<0.001	0.99 (0.99–0.99)	<0.001
Albumin	0.88 (0.87–0.88)	<0.001	0.95 (0.94–0.96)	<0.001
C-reactive protein	1.10 (1.10–1.11)	<0.001	1.07 (1.06–1.08)	<0.001
D-dimer (for every 1,000 ng/ml rise)	1.22 (1.20–1.23)	<0.001	1.03 (1.01–1.06)	0.008

COVID-19, coronavirus disease-2019; CI, confidence interval; SARS-CoV-2, severe acute respiratory syndrome coronavirus 2; RT-PCR, reverse transcription polymerase chain reaction; Ct value, cycle threshold value; COAD, chronic obstructive airway disease; eGFR, estimated glomerular filtration rate; CKD-EPI, chronic kidney disease epidemiology collaboration.

As for non-hospitalized patient, 550 patients (2.7%) died within 90 days, of which 194 and 237 patients were hypo- and normonatremic at presentation. Non-hospitalized hyponatremic patients had a lower 90-day survival rate (96.8 vs. 98.2%, *p* < 0.001). In the multivariate analysis, severe hyponatremia predicted 90-day survival after adjusting for confounders (aHR 2.25, 95% CI 1.16–4.35, *P* = 0.017).

### Comparison with SARS

Data from 1,516 SARS adult patients were retrieved for comparison with patients with COVID-19 (Supplementary Figure 2). Hospitalized patients with COVID-19 were older than those with SARS (64.7 ± 21.9 years vs. 47.0 ± 19.1 years, *P* < 0.001). The absolute rate of hyponatremia was higher in SARS patients (44.0 vs. 25.4%, *P* < 0.001). 132/667 (20%) of SARS patients with hyponatremia died compared to 182/849 (14.1%) of non-hyponatremic patients (Figure 2, *P* < 0.001), but this was not statistically significant after adjusting for gender, age and other biochemical parameters. A similar percentage of SARS patients requiring ICU care presented with hyponatremia compared to other SARS patients (42.3 vs. 43.2%, *P* = 0.10).

### Relationship of local waves of COVID-19 and hyponatremia

Patients presenting during the “fifth wave” had the highest rate of hyponatremia at presentation (Table 2). This difference was observed in all analyzed subgroups, including the elderly (Supplementary Figure 3). Compared to previous waves in Hong Kong, hyponatremic patients during the “fifth wave” were older and had more medical comorbidities (Supplementary Table 6). Multivariate analysis showed that patients during the “fifth wave” had a 2.29-fold risk (95% CI 2.02–2.59, *P* < 0.001) of presenting with hyponatremia (Supplementary Table 7).

## Discussion

COVID-19 is a systemic disease with various clinical manifestations in multiple organ systems. In this territory-wide retrospective analysis, around a quarter of adult patients with COVID-19 had hyponatremia of any degree. The importance of hyponatremia is underscored by its correlation with mortality and ICU admission, suggesting that hyponatremia may be a cardinal feature and prognostic indicator of moderate-to-severe COVID-19.

The rate of hyponatremia appeared to be particularly high during the “fifth wave” in Hong Kong involving the Omicron BA.2 subvariant. Up to 31.7% of patients in the “fifth wave” were found to be hyponatremic, compared to only 7.3% of patients during the “second wave” predominated by the original SARS-CoV-2 virus (*P* < 0.001). While it is likely that different variants of SARS-CoV-2 are associated with distinct disease presentations, patient characteristics may have played a role as well. During earlier outbreaks in Hong Kong, the territory followed a strict policy of “zero COVID,” and all patients and contacts were rigorously traced, diagnosed by RT-PCR, and hospitalized (30). In contrast, admitting all infected patients was logistically impossible during the large-scale Omicron outbreak. Many infected patients did not present to health care services, and fewer still had laboratory investigations performed. The patient populations of earlier waves may have captured a disproportionately large percentage of patients with asymptomatic or mild disease, resulting in lower rates of hyponatremia. During the “fifth wave,” hyponatremic patients in our analysis were significantly older and had more medical comorbidities than previous waves, suggesting the possibility of selection bias (Supplementary Table 6).

Our analysis shows that both disease-specific factors and patient factors modulate the risk of hyponatremia in COVID-19. Importantly, our study is the first to demonstrate a correlation between viral load of SARS-CoV-2, as demonstrated by the Ct value from respiratory specimens, and hyponatremia. Adding to that, CRP, an end-product of the IL-6 inflammatory pathway, was tied with an increased risk of hyponatremia. This is consistent with previous studies correlating IL-6 levels with hyponatremia in COVID-19 and other inflammatory conditions, possibly through stimulation of pituitary ADH secretion (21, 22). These findings suggest that SARS-CoV-2 viral antigens may directly contribute to the pathogenesis of hyponatremia *via* activation of inflammatory pathways.

Patients who presented with hyponatremia tended to be older, male, and with more medical comorbidities. Although these same factors are also recognized risk factors for developing moderate-to-severe disease in COVID-19, multivariate analysis showed that certain comorbidities such as DM, active malignancy, CHF, dementia and CKD may particularly predispose patients to develop hyponatremia, independent of other risk factors and indices of systemic inflammation (31, 32). The mechanisms by which these conditions contribute to hyponatremia in COVID-19 remain speculative, with a paucity of reliable biochemical and clinical data available from large registry studies to explain the underlying pathophysiology.

The overall mortality of COVID-19 patients with hyponatremia was high, and was significantly greater than those without hyponatremia. Thirty-day mortality was up to 9.7% for hyponatremic patients compared to 5.7% for normonatremic patients. Regression analysis showed that the excess risk for death was primarily driven by patients with moderate and severe hyponatremia. Furthermore, deaths associated with hyponatremia tended to occur early, and there was little difference between the mortality rate at 30 and 90 days. Hyponatremia was also a predictor of ICU admission, especially with more severe degrees of hyponatremia. Paradoxically, patients with severe hyponatremia actually had shorter ICU stays than normonatremic patients, likely due to the extremely high rates of early mortality among patients with severe hyponatremia. In general, hyponatremic patients had longer hospital stays than normonatremic ones, which could be due to greater severity of disease as well as the need for close monitoring to avoid over-rapid correction of hyponatremia. All these factors conspire to burden heavily overstretched healthcare systems during large-scale COVID-19 outbreaks.

In our study, we further compared the prevalence and outcomes of hyponatremia among patients with COVID-19 with their counterparts with SARS during the 2003 outbreak of SARS-CoV-1. This comparison is salient, as the SARS-CoV-1 pandemic was the most widespread and significant coronavirus outbreak before the current COVID-19 pandemic, and the two causative viruses share significant homology (33). In our analysis, hyponatremia was not a significant predictor for either mortality or ICU admission during the SARS-CoV-1 outbreak after adjusting for other demographic variables and biochemical parameters, contrasting with the strong dose-response relationship for mortality noted in COVID-19. These findings suggest that respiratory tract infections due to different coronaviruses, including different variants of the same species, may manifest different clinical and biochemical phenotypes. These findings are analogous to the differing rates of hyponatremia with different causes of bacterial pneumonia, with higher rates observed in atypical infections such as legionellosis (24, 25).

This study has certain limitations. First, paired plasma and urine biochemistries were unavailable for many patients with hyponatremia. Volume status could not be accurately ascertained and the etiology of hyponatremia in many cases could not be determined with confidence. To overcome this, we attempted to evaluate the volume status by the percentage change of serum creatinine from hyponatremia to eunatremia. Second, this study was observational in nature. Without specific interventions directed at correcting hyponatremia, a direct causal effect of hyponatremia on clinical outcomes could not be established. Third, morbidity from severe hyponatremia often arises from inappropriate correction of hyponatremia. However, the method of correction of hyponatremia, including solute therapy, fluid restriction, saline infusions, or other pharmacological interventions, was not available from our registry analysis. Fourth, we also used the measured plasma sodium before adjusting for glucose level in the analysis. In addition, the measurement of plasma sodium by the indirect ion selective electrode (ISE) methods may further be affected when the plasma glucose reaches an exceedingly high level (34, 35). Fifth, the time lag between onset of symptoms and presentation to health care services could not be accounted for. Delayed presentation may have contributed to a higher prevalence of hyponatremia and severe disease, although these discrepancies also reflect real-world variations in healthcare access and evolutions in health policy. Sixth, due to the limitation of the registry, the disease coding is based in ICD-9, but not the most updated ICD-10. Nevertheless, this database has a high diagnostic accuracy, and has been widely used in several publications (36, 37). Finally, we could not exclude an impact on the prevalence and outcomes of hyponatremia related to the availability of COVID-19 vaccines and use of immunomodulatory or antiviral treatments in later waves. In Hong Kong, in earlier waves of COVID-19, treatment consisted of combinations of interferon-1β, lopinavir-ritonavir, ribavirin, and corticosteroids; treatment evolved to encompass antivirals including remdesivir, molnupiravir and nirmatrelvir-ritonavir, and novel immunomodulatory agents including baricitinib and tocilizumab in later waves. Crucially, hyponatremia was a convincing risk factor for mortality and other adverse outcomes regardless of the wave of COVID-19 and the availability of COVID-19 treatments.

A key strength of our study is the large number of patients included in this territory-wide analysis, all of who were diagnosed by RT-PCR. Data on viral loads were available for all patients, allowing for correlation between viral burden and important clinical parameters, including hyponatremia. All patients with COVID-19 were followed up for at least 90 days or until death, and received similar standards of medical care, providing insight into real-world outcomes. Furthermore, our analysis included comparisons across different waves of COVID-19 locally, each attributable to a different circulating variant, as well as a comparative analysis with a historical cohort of patients with SARS-CoV-1 infection. This allowed for the characterization of a syndrome of hyponatremia specific for COVID-19, particularly for the dominant Omicron BA.2 subvariant during the largest local outbreak thus far.

## Conclusion

Hyponatremia is common among COVID-19 patients, and is associated with adverse outcomes including mortality, prolonged hospitalization, and ICU admission. Further research is required to clarify the causes of hyponatremia in COVID-19, and identify appropriate strategies for the treatment of hyponatremia and optimization of clinical outcomes in COVID-19.

## Data availability statement

The raw data supporting the conclusions of this article will be made available by the authors, without undue reservation.

## Ethics statement

The studies involving human participants were reviewed and approved by Institutional Review Board of the University of Hong Kong/Hospital Authority Hong Kong West Cluster, Hong Kong SAR, China. Written informed consent for participation was not required for this study in accordance with the national legislation and the institutional requirements.

## Author contributions

GC, CW, BS, KC, and DY conceptualized the work. GC, CW, and BS drafted the original manuscript. JN, GL, IH, HT, ST, TC, KC, and DY reviewed and edited the manuscript. All authors contributed to the article and approved the submitted version.
