# Supplementary material for: Epidemiology and outcomes of hyponatremia in patients with COVID-19—A territory-wide study in Hong Kong
Source: Front Med (Lausanne). 2023 Jan 11;9:1096165. doi: 10.3389/fmed.2022.1096165 (PMC9874105; doi:10.3389/fmed.2022.1096165)

# SUPPLEMENTAL MATERIAL

**Supplemental Table 1. ICD-9 diagnostic code used for data retrieval in the Clinical Data Analysis and Reporting System (CDARS) database of the Hong Kong Hospital Authority**

**Supplemental Table 2. Time period and dominant SARS-CoV-2 variants in each local wave during COVID-19**

**Supplemental Table 3. Clinical characteristics of COVID-19 patients who required intensive care unit admission**

**Supplemental Table 4. The length of stay and estimated costs during general medical ward and intensive care unit admission in COVID-19 patients**

**Supplemental Table 5. Clinical characteristics of COVID-19 patients who died within 30 days**

**Supplemental Table 6. Clinical characteristics of COVID-19 patients with hyponatremia at presentation during the different local waves**

**Supplemental Table 7. Risk factors for hyponatremia in patients with COVID-19**

**Supplemental Figure 1. 90-day mortality in (A) COVID and (B) SARS patients with mild, moderate-severe hyponatremia and normonatremia.**

**Supplemental Figure 2. Flowchart of SARS patients included in study**

**Supplemental Figure 3. Hyponatremia in different local waves of COVID-19**

**Supplemental Table 1. ICD-9 diagnostic code used for data retrieval in the Clinical Data Analysis and Reporting System (CDARS) database of the Hong Kong Hospital Authority**

| Coronavirus disease-2019 (COVID-19) | 079.89: Infection due to coronavirus  480.8 (1): Pneumonia due to coronavirus  519.8 (8): COVID-19 |
| --- | --- |
| Severe Acute Respiratory Syndrome (SARS) | 465.9 (2): Severe acute respiratory syndrome involving upper respiratory tract  466.0 (1): Severe acute respiratory syndrome with acute bronchitis  480.0 (1): Pneumonia due to coronavirus  480.8 (2): Severe acute respiratory syndrome with atypical pneumonia  480.8 (4): Severe acute respiratory syndrome  480.8 (5): Severe acute respiratory syndrome  079.89 (3): Infection due to coronavirus |
| Diabetes mellitus | 250: Diabetes mellitus |
| Hypertension | 401 to 405: Hypertensive disease |
| Ischemic heart disease | 410 to 414: Ischemic heart disease |
| Cerebrovascular accident | 430 to 438: Cerebrovascular disease |
| Cardiac arrhythmia | 426: Conduction Disorders  427: Cardiac dysrhythmias |
| Congestive heart failure | 428: Heart failure |
| COAD | 491: Chronic bronchitis  492: Emphysema  494: Bronchiectasis  496: Chronic airway obstruction, not elsewhere classified |
| Asthma | 493: Asthma |
| Pneumoconiosis | 500 to 508: Pneumoconiosis |
| Dementia | 290: Senile and presenile organic psychotic conditions  294.1: Dementia in conditions classified elsewhere  331: Other cerebral degenerations |
| Chronic liver disease | 571: Chronic liver disease and cirrhosis  572.3: Portal hypertension  572.4: Hepatorenal syndrome  573: Other disorders of liver |
| Active malignancy | 140 to 239: Neoplasms |

**Supplemental Table 2. Time period and dominant SARS-CoV-2 variants in each local wave during COVID-19**

| **Wave** | **Time period** | **Dominant causative SARS-CoV-2 variants** |
| --- | --- | --- |
| Second | 1 – 30 April 2020 | D614G (31) |
| Third | 15 June – 30 September 2020 | B.1.1.63 (32) |
| Fourth | 1 November 2022 – 28 February 2021 | B.1.36.27 (32) |
| Fifth | 1 January – 31 March 2022 | Omicron BA.2 |

**Supplemental Table 3. Clinical characteristics of COVID-19 patients who required intensive care unit admission**

|  | **Required ICU care**  **(n = 1,609)** | **Without ICU care**  **(n = 31,068)** | **P-value** |
| --- | --- | --- | --- |
| Age | 66.5 ± 14.4 | 64.6 ± 22.4 | 0.001^a^ |
| Age older than 65, No. (%) | 939 (58.4%) | 16,717 (53.8%) | < 0.001^b^ |
| Male, No. (%) | 1,040 (64.6%) | 15,877 (51.1%) | < 0.001^b^ |
| Serum sodium (mmol/L) | 133.6 ± 9.0 | 137.3 ± 6.4 | < 0.001^a^ |
| Hyponatremia, No. (%) | 732 (45.5%) | 7,460 (24.0%) | < 0.001^b^ |
| Mild | 442 (27.5%) | 5,088 (16.4%) |  |
| Moderate | 120 (7.5%) | 1,426 (4.6%) |  |
| Severe | 170 (10.6%) | 946 (3.0%) |  |
| **Comorbidities** | | | |
| Diabetes mellitus | 581 (36.1%) | 6,999 (22.5%) | < 0.001^b^ |
| Hypertension | 821 (51.0%) | 12,627 (40.6%) | < 0.001^b^ |
| Ischemic heart disease | 247 (15.4%) | 3,453 (11.1%) | < 0.001^b^ |
| Cerebrovascular accident | 126 (7.8%) | 2,816 (9.1%) | 0.09^b^ |
| Cardiac arrhythmia | 245 (15.2%) | 3,594 (11.6%) | < 0.001^b^ |
| Congestive heart failure | 176 (10.9%) | 2,509 (8.1%) | < 0.001^b^ |
| Chronic obstructive airway disease | 77 (4.8%) | 1,480 (4.8%) | 1.0^b^ |
| Asthma | 27 (1.7%) | 507 (1.6%) | 0.9^b^ |
| Pneumoconiosis | 15 (0.9%) | 288 (0.9%) | 1.0^b^ |
| Dementia | 50 (3.1%) | 3,391 (10.9%) | < 0.001^b^ |
| Chronic liver disease | 149 (9.3%) | 1,762 (5.7%) | < 0.001^b^ |
| Active malignancy | 357 (22.2%) | 4,537 (14.6%) | < 0.001^b^ |
| Chronic kidney disease | | | < 0.001^b^ |
| Stage 1 | 388 (24.1%) | 10,231 (32.9%) |  |
| Stage 2 | 618 (38.4%) | 13,315 (42.9%) |  |
| Stage 3 | 321 (20.0%) | 4,534 (14.6%) |  |
| Stage 4 | 111 (6.9%) | 1,670 (5.4%) |  |
| Stage 5 | 171 (10.6%) | 1,318 (4.2%) |  |

Data are presented as mean ± standard deviation unless specified and compared by Student’s t-test^a^ and chi-square test^b^.

SARS, Severe Acute Respiratory Syndrome; COVID-19, coronavirus disease-2019; SARS-CoV-2, severe acute respiratory syndrome coronavirus 2; RT-PCR, reverse transcription polymerase chain reaction; Ct value, cycle threshold value

**Supplemental Table 4. The length of stay during general medical ward and intensive care unit admission in COVID-19 patients**

|  | **Length of stay* (Days)**  **Median (IQR)** | **p-value^#^** |
| --- | --- | --- |
| **General Medical Ward** |  |  |
|  |  |  |
| No hyponatremia | 5.0 (1.5 - 8.5) | - |
| Hyponatremia | 6.0 (2.5 - 9.5) | <0.001* |
| Mild hyponatremia | 6.0 (2.5 - 9.5) | <0.001* |
| Moderate hyponatremia | 6.0 (2.5 - 9.5) | <0.001* |
| Severe hyponatremia | 6.0 (3.0 - 9.0) | 0.02* |
| **Intensive Care Unit** |  |  |
|  |  |  |
| No hyponatremia | 5.0 (1.0 - 9.0) | - |
| Hyponatremia | 4.0 (1.0 - 7.0) | 0.06 |
| Mild hyponatremia | 5.0 (1.0 - 9.0) | 0.79 |
| Moderate hyponatremia | 3.5 (1.0 - 6.0) | 0.07 |
| Severe hyponatremia | 3.0 (1.5 - 4.5) | <0.001* |

^#^Whitney U test comparing against no hyponatremia; Abbreviations: IQR, interquartile range.

**Supplemental Table 5. Clinical characteristics of COVID-19 patients who died within 30 days**

|  | **Died**  **(n = 4,318)** | **Survived**  **(n = 49,097)** | **P-value** |
| --- | --- | --- | --- |
| Age | 83.2 ± 11.5 | 65.4 ± 21.3 | < 0.001^a^ |
| Age older than 65, No. (%) | 3,979 (92.1%) | 27,136 (55.3%) | < 0.001^b^ |
| Male, No. (%) | 2,596 (60.1%) | 25,044 (51.0%) | < 0.001^b^ |
| Serum sodium (mmol/L) | 138.7 ± 10.2 | 136.8 ± 6.1 | < 0.001^a^ |
| Hyponatremia, No. (%) | 1,407 (32.6%) | 13,138 (26.8%) | < 0.001^b^ |
| Mild | 787 (18.2%) | 9,026 (18.4%) |  |
| Moderate | 362 (8.4%) | 2,459 (5.0%) |  |
| Severe | 258 (5.9%) | 1,653 (3.4%) |  |
| **Comorbidities** | | | |
| Diabetes mellitus | 1,470 (34.0%) | 11,475 (23.4%) | < 0.001^b^ |
| Hypertension | 2,707 (62.7%) | 20,512 (41.8%) | < 0.001^b^ |
| Ischemic heart disease | 859 (19.9%) | 5,562 (11.3%) | < 0.001^b^ |
| Cerebrovascular accident | 822 (19.0%) | 4,395 (9.0%) | < 0.001^b^ |
| Cardiac arrhythmia | 986 (22.8%) | 5,610 (11.4%) | < 0.001^b^ |
| Congestive heart failure | 760 (17.6%) | 3,755 (7.6%) | < 0.001^b^ |
| Chronic obstructive airway disease | 414 (9.6%) | 2,303 (4.7%) | < 0.001^b^ |
| Asthma | 72 (1.7%) | 850 (1.7%) | 0.8^b^ |
| Pneumoconiosis | 111 (2.6%) | 370 (0.8%) | 0.001^b^ |
| Dementia | 1,093 (25.3%) | 5,091 (10.4%) | < 0.001^b^ |
| Chronic liver disease | 376 (8.7%) | 2,891 (5.9%) | < 0.001^b^ |
| Active malignancy | 905 (21.0%) | 8,090 (16.5%) | < 0.001^b^ |
| Chronic kidney disease | | | < 0.001^b^ |
| Stage 1 | 244 (5.7%) | 14,401 (29.3%) |  |
| Stage 2 | 1,513 (35.0%) | 23,548 (48.0%) |  |
| Stage 3 | 1,192 (27.6%) | 7,252 (14.8%) |  |
| Stage 4 | 762 (17.6%) | 2,205 (4.5%) |  |
| Stage 5 | 607 (14.1%) | 1,691 (3.4%) |  |

Data are presented as mean ± standard deviation unless specified and compared by Student’s t-test^a^ and chi-square test^b^.

SARS, Severe Acute Respiratory Syndrome; COVID-19, coronavirus disease-2019; SARS-CoV, severe acute respiratory syndrome coronavirus.

**Supplemental Table 6. Clinical characteristics of COVID-19 patients with hyponatremia at presentation during the different local waves**

|  | **2^nd^ wave**  **(n = 59)** | **3^rd^ wave**  **(n = 309)** | **4^th^ wave**  **(n = 562)** | **5^th^ wave**  **(n = 13,530)** | **P-value** |
| --- | --- | --- | --- | --- | --- |
| Age | 52.5 ± 13.8 | 63.5 ± 15.5 | 62.3 ± 14.5 | 75.3 ± 15.8 | < 0.001 ^a^ |
| Age older than 65, No. (%) | 7 (11.9%) | 151 (48.9%) | 233 (41.5%) | 10,469 (77.4%) | < 0.001 ^b^ |
| Male, No. (%) | 50 (84.7%) | 191 (61.8%) | 317 (56.4%) | 7,626 (56.4%) | < 0.001 ^b^ |
| Serum sodium (mmol/L) | 132.2 ± 2.6 | 132.2 ± 2.7 | 132.3 ± 2.4 | 129.7 ± 5.8 | < 0.001 ^a^ |
| Hyponatremia, No. (%) | 59 (7.3%) | 309 (9.9%) | 562 (11.1%) | 13,530 (33.8%) | < 0.001 ^b^ |
| Mild | 50 (6.2%) | 267 (8.5%) | 499 (9.8%) | 8,921 (20.9%) |  |
| Moderate | 7 (0.9%) | 34 (1.1%) | 53 (1.0%) | 2,720 (6.4%) |  |
| Severe | 2 (0.2%) | 8 (0.3%) | 10 (0.2%) | 1,889 (4.4%) |  |
| **Comorbidities, No. (%)** | | | | |  |
| Diabetes mellitus | 13 (22.0%) | 105 (34.0%) | 173 (30.8%) | 4,655 (34.4%) | 0.05 ^b^ |
| Hypertension | 9 (15.3%) | 137 (44.3%) | 230 (40.9%) | 7,521 (55.6%) | < 0.001 ^b^ |
| Ischemic heart disease | 3 (5.1%) | 32 (10.4%) | 46 (8.2%) | 2,160 (16.0%) | < 0.001 ^b^ |
| Cerebrovascular accident | 1 (1.7%) | 16 (5.2%) | 27 (4.8%) | 1,780 (13.2%) | < 0.001 ^b^ |
| Cardiac arrhythmia | 0 (0%) | 33 (10.7%) | 36 (6.4%) | 2,170 (16.0%) | < 0.001 ^b^ |
| Congestive heart failure | 0 (0%) | 15 (4.9%) | 16 (2.8%) | 1,441 (10.7%) | < 0.001 ^b^ |
| Chronic obstructive airway disease | 2 (3.4%) | 7 (2.3%) | 8 (1.4%) | 937 (6.9%) | < 0.001 ^b^ |
| Asthma | 0 (0%) | 2 (0.6%) | 7 (1.2%) | 270 (2.0%) | 0.1 ^b^ |
| Pneumoconiosis | 0 (0%) | 6 (1.9%) | 3 (0.5%) | 192 (1.4%) | 0.2 ^b^ |
| Dementia | 0 (0%) | 12 (3.9%) | 9 (1.6%) | 1,710 (12.6%) | < 0.001 ^b^ |
| Chronic liver disease | 2 (3.4%) | 34 (11.0%) | 32 (5.7%) | 960 (7.1%) | 0.04 ^b^ |
| Active malignancy | 5 (8.5%) | 38 (12.3%) | 68 (12.1%) | 2,890 (21.4%) | < 0.001 ^b^ |
| **Chronic kidney disease (eGFR below 90)** | 26 (44.1%) | 190 (61.5%) | 330 (58.7%) | 11,420 (84.4%) | < 0.001 ^b^ |
| eGFR between 60 and 90 (Stage 2) | 24 (40.7%) | 123 (39.8%) | 239 (42.5%) | 6,885 (50.9%) |  |
| eGFR between 30 and 60 (Stage 3) | 2 (3.4%) | 52 (16.8%) | 74 (13.2%) | 2,737 (20.2%) |  |
| eGFR between 15 and 30 (Stage 4) | 0 (0%) | 10 (3.2%) | 11 (2.0%) | 893 (6.6%) |  |
| eGFR below 15 (Stage 5) | 0 (0%) | 5 (1.6%) | 6 (1.1%) | 905 (6.7%) |  |
| **Laboratory parameters** | | | | |  |
| SARS-CoV-2 RT-PCR Ct value | 26.7 ± 4.5 | 23.0 ± 5.4 | 23.9 ± 5.4 | 22.2 ± 6.4 | < 0.001 ^a^ |
| Hemoglobin (g/dL) | 14.1 ± 1.4 | 13.2 ± 1.8 | 13.2 ± 1.7 | 11.8 ± 2.2 | < 0.001 ^a^ |
| White cell count (10^9^/L) | 5.9 ± 3.0 | 5.9 ± 2.4 | 5.7 ± 2.3 | 9.0 ± 7.2 | < 0.001 ^a^ |
| Neutrophil (10^9^/L) | 4.2 ± 2.6 | 4.4 ± 2.5 | 4.1 ± 2.2 | 6.7 ± 4.8 | < 0.001 ^a^ |
| Lymphocyte (10^9^/L) | 1.1 ± 0.4 | 1.1 ± 0.5 | 1.1 ± 0.6 | 1.0 ± 2.0 | 0.7 ^a^ |
| Neutrophil to lymphocyte ratio | 4.5 ± 3.4 | 5.4 ± 4.9 | 4.9 ± 5.4 | 9.5 ± 10.5 | < 0.001 ^a^ |
| Platelet (10^9^/L) | 230 ± 74 | 221 ± 76 | 219 ± 69 | 222 ± 97 | < 0.001 ^a^ |
| Potassium (mmol/L) | 3.9 ± 0.4 | 3.8 ± 0.4 | 3.8 ± 0.4 | 4.0 ± 0.6 | < 0.001 ^a^ |
| Urea (mmol/L) | 4.3 ± 1.1 | 5.7 ± 3.9 | 5.4 ± 3.7 | 9.1 ± 8.2 | < 0.001 ^a^ |
| Creatinine (umol/L) | 82.0 ± 16.7 | 94.0 ± 84.2 | 88.9 ± 93.8 | 142.3 ± 186.5 | < 0.001 ^a^ |
| eGFR (by CKD-EPI) | 90.4 ± 17.6 | 78.6 ± 24.0 | 82.1 ± 23.0 | 65.8 ± 27.7 | < 0.001 ^a^ |
| Albumin (g/L) | 37.7 ± 6.2 | 35.8 ± 6.0 | 36.3 ± 5.3 | 32.6 ± 6.4 | < 0.001 ^a^ |
| C-reactive protein (mg/L) | 5.3 ± 6.9 | 5.2 ± 5.3 | 4.1 ± 4.6 | 6.9 ± 7.3 | < 0.001 ^a^ |
| Calcium (mmol/L) | 2.30 ± 0.11 | 2.24 ± 0.12 | 2.24 ± 0.12 | 2.21 ± 0.17 | < 0.001 ^a^ |
| Phosphate (mmol/L) | 1.05 ± 0.20 | 1.02 ± 0.21 | 1.03 ± 0.21 | 1.12 ± 0.46 | < 0.001 ^a^ |
| Osmolality (mOsm/kg) | 263 ± 7 | 273 ± 16 | 272 ± 12 | 269 ± 23 | 0.3 ^a^ |
| Thyroid stimulating hormone (mIU/L) | 1.4 ± 1.2 | 1.3 ± 1.2 | 1.4 ± 1.9 | 1.6 ± 3.8 | 0.3 ^a^ |
| D-dimer (ng/ml) | 725 ± 881 | 622 ± 829 | 533 ± 795 | 1326 ± 1906 | < 0.001 ^a^ |
| Urine sodium (mmol/L) | 45.2 ± 64.7 | 44.8 ± 26.5 | 44.4 ± 27.1 | 49.5 ± 34.9 | 0.09 ^a^ |
| Urine osmolality | 463 ± 247 | 427 ± 188 | 459 ± 193 | 403 ± 161 | 0.008 ^a^ |

Data are presented as mean ± standard deviation unless specified and compared by Student’s t-test^a^ and chi-square test^b^.

SARS-CoV-2, severe acute respiratory syndrome coronavirus 2; RT-PCR, reverse transcription polymerase chain reaction; Ct value, cycle threshold value; eGFR, estimated glomerular filtration rate; CKD-EPI, Chronic Kidney Disease Epidemiology Collaboration.

**Supplemental Table 7. Risk factors for hyponatremia in patients with COVID-19**

|  | **Univariate model** | | **Multivariate model** | |
| --- | --- | --- | --- | --- |
|  | **OR (95% CI)** | **P-value** | **OR (95% CI)** | **P-value** |
| Age | 1.03 (1.03 – 1.03) | < 0.001 | 1.02 (1.01 – 1.02) | < 0.001 |
| Male sex | 1.30 (1.26 – 1.36) | < 0.001 | 1.15 (1.07 – 1.24) | < 0.001 |
| **Comorbidities** | | | | |
| Diabetes mellitus | 2.09 (2.01 – 2.19) | < 0.001 | 1.39 (1.28 – 1.51) | < 0.001 |
| Active malignancy | 1.45 (1.38 – 1.52) | < 0.001 | 1.17 (1.07 – 1.29) | 0.001 |
| Dementia | 1.31 (1.23 – 1.40) | < 0.001 | 0.63 (0.56 – 0.70) | < 0.001 |
| Congestive heart failure | 1.41 (1.32 – 1.51) | < 0.001 | 0.74 (0.65 – 0.84) | < 0.001 |
| **Laboratory parameters** | | | | |
| White cell count | 1.08 (1.08 – 1.09) | < 0.001 | 1.03 (1.03 – 1.04) | < 0.001 |
| Creatinine (every 100 µmol/L rise) | 1.17 (1.15 – 1.19) | < 0.001 | 1.04 (1.02 – 1.07) | < 0.001 |
| Albumin | 0.92 (0.91 – 0.92) | < 0.001 | 0.97 (0.96 – 0.97) | < 0.001 |
| C-reactive protein | 1.07 (1.07 – 1.07) | < 0.001 | 1.01 (1.01 – 1.02) | < 0.001 |
| SARS-CoV-2 PCR Ct value | 0.97 (0.97 – 0.98) | < 0.001 | 0.98 (0.98 – 0.99) | < 0.001 |
| Local 5^th^ wave outbreak (Compared to other waves) | 4.88 (4.56 – 5.22) | < 0.001 | 2.29 (2.02 – 2.59) | < 0.001 |

COVID-19, coronavirus disease-2019; CI, confidence interval; SARS-CoV, severe acute respiratory syndrome coronavirus; RT-PCR, reverse transcription polymerase chain reaction; Ct value, cycle threshold value

**Supplemental Figure 1. 90-day mortality in (A) COVID and (B) SARS patients with mild, moderate-severe hyponatremia and normonatremia.**

**
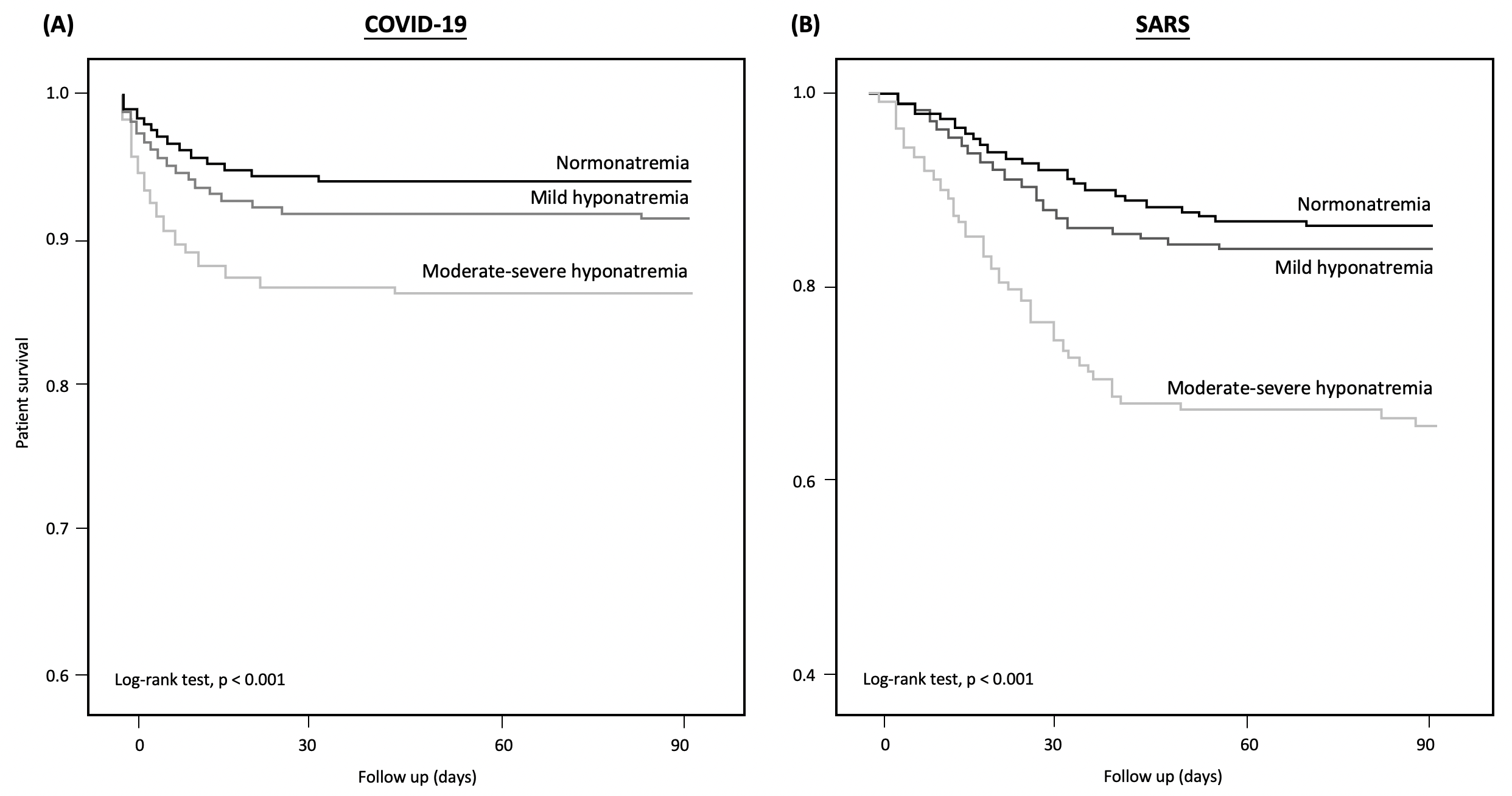
**

**Supplemental Figure 2. Flowchart of SARS patients included in study**


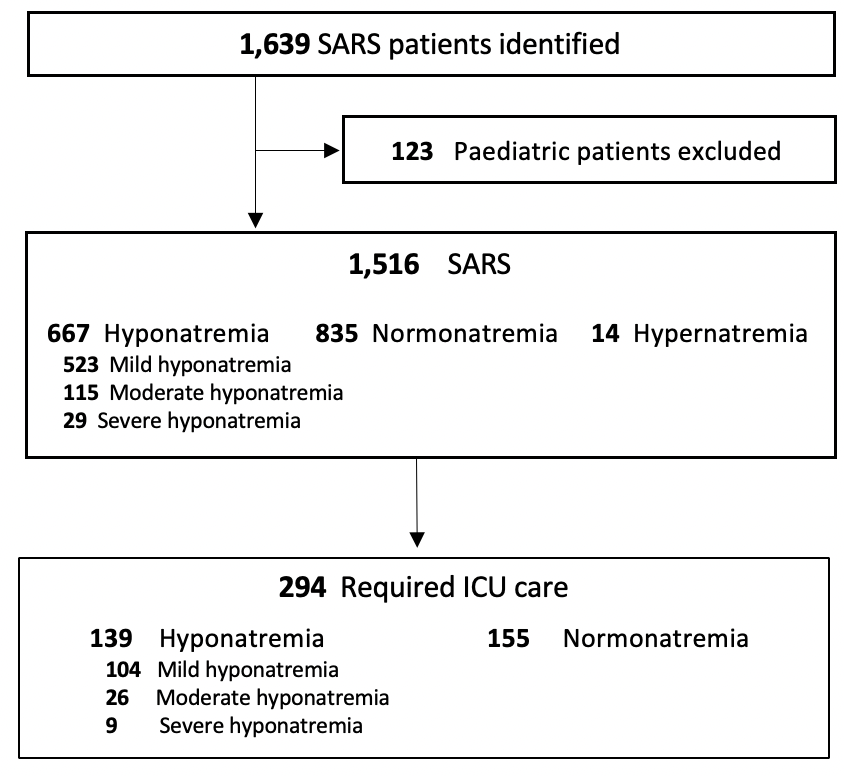


**Supplemental Figure 3. Hyponatremia in different local waves of COVID-19**


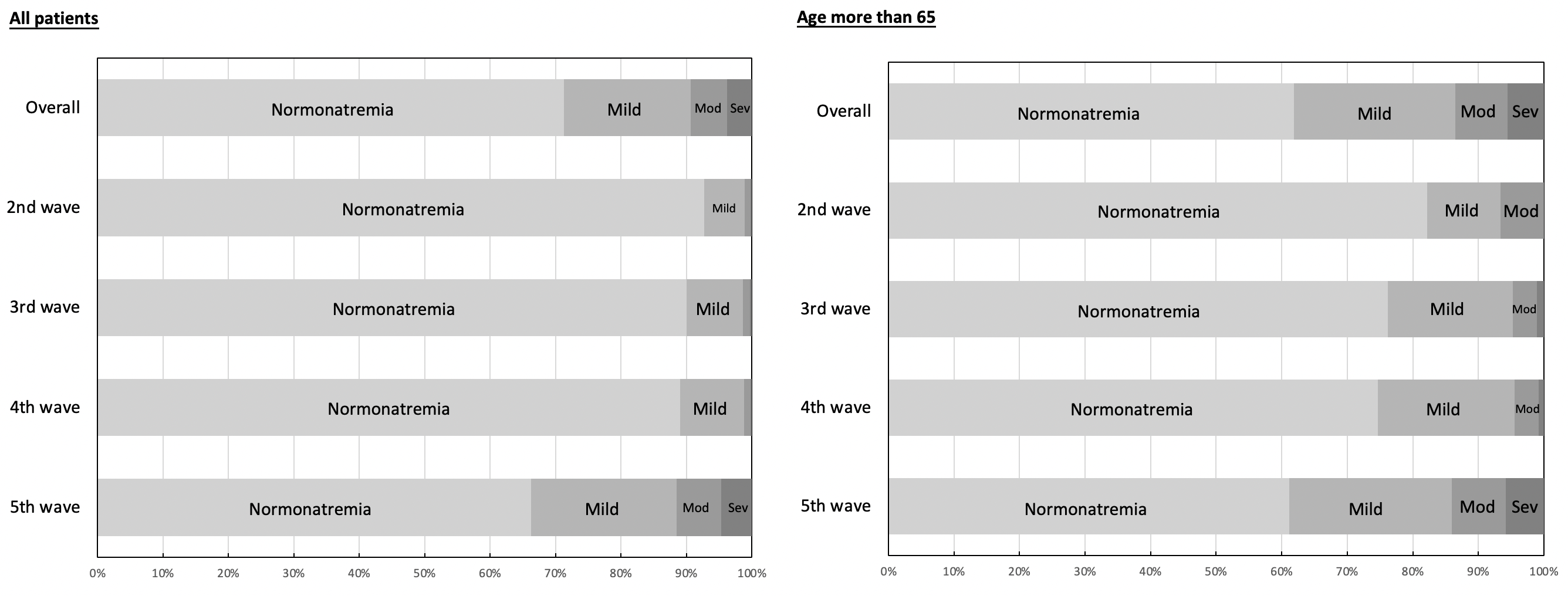

Supplement: Supplementary file 1 [file Data_Sheet_1.docx]
